# Sugarcane/soybean intercropping with reduced nitrogen addition promotes photosynthesized carbon sequestration in the soil

**DOI:** 10.3389/fpls.2023.1282083

**Published:** 2023-12-01

**Authors:** Tantan Zhang, Hu Tang, Peng Peng, Shiqiang Ge, Yali Liu, Yuanjiao Feng, Jianwu Wang

**Affiliations:** ^1^ Key Laboratory of Agro-Environments in Tropics, Ministry of Agriculture and Rural Affairs, South China Agriculture University, Guangzhou, China; ^2^ Guangdong Provincial Key Laboratory of Eco-Circular Agriculture, South China Agriculture University, Guangzhou, China; ^3^ Department of Ecology, College of Natural Resources and Environment, South China Agricultural University, Guangzhou, China

**Keywords:** sugarcane/soybean intercropping, 13C pulse labelling, C cycle, soil biochemistry, root growth

## Abstract

**Introduction:**

Sugarcane/soybean intercropping with reduced nitrogen (N) addition has improved soil fertility and sustainable agricultural development in China. However, the effects of intercropping pattern and N fertilizer addition on the allocation of photosynthesized carbon (C) in plant-soil system were far less understood.

**Methods:**

In this study, we performed an ^13^CO_2_ pulse labeling experiment to trace C footprints in plant-soil system under different cropping patterns [sugarcane monoculture (MS), sugarcane/soybean intercropping (SB)] and N addition levels [reduced N addition (N1) and conventional N addition (N2)].

**Results and discussion:**

Our results showed that compared to sugarcane monoculture, sugarcane/soybean intercropping with N reduced addition increased sugarcane biomass and root/shoot ratio, which in turn led to 23.48% increase in total root biomass. The higher root biomass facilitated the flow of shoot fixed ^13^C to the soil in the form of rhizodeposits. More than 40% of the retained ^13^C in the soil was incorporated into the labile C pool [microbial biomass C (MBC) and dissolved organic C (DOC)] on day 1 after labeling. On day 27 after labeling, sugarcane/soybean intercropping with N reduced addition showed the highest ^13^C content in the MBC as well as in the soil, 1.89 and 1.14 times higher than the sugarcane monoculture, respectively. Moreover, intercropping pattern increased the content of labile C and labile N (alkaline N, ammonium N and nitrate N) in the soil. The structural equation model indicated that the cropping pattern regulated ^13^C sequestration in the soil mainly by driving changes in labile C, labile N content and root biomass in the soil. Our findings demonstrate that sugarcane/soybean intercropping with reduced N addition increases photosynthesized C sequestration in the soil, enhances the C sink capacity of agroecosystems.

## 1 Introduction

Small changes in soil, as the largest organic C pool in terrestrial ecosystems, may cause dramatic changes in atmospheric CO_2_ concentrations, so determining the dynamics of soil C sequestration plays a key role in understanding the global C balance (Lim et al., 2020). In addition to plant residues, rhizodeposits are a key source of photosynthesized C input into the soil during plant growth and play a linking role in the continuous soil-plant-atmosphere C cycle (Bowsher et al., 2018). Rhizodeposits are largely composed of root exudates, secretions, mycorrhizal hyphae, sloughed-off root cells, and senescing roots (Leake et al., 2006). Rhizodeposit transformation dominates the rhizosphere C flow, provides the required energy for the soil microbial community, shapes its structure and function, and drives different C sequestration processes (Cesarz et al., 2013; Li et al., 2022b). Most primary production is fixed in the soil through microbial biomass, so soil microbial biomass can be used to quantify the flow of rhizodeposits to better understand the C cycle in the soil (Jin et al., 2013).

Currently, pulse labelling by short-term exposure to ^13^CO_2_ is the most commonly used method to analyze photosynthesized C input and allocation in plant-soil systems (Studer et al., 2014; Zhu et al., 2017b). This method has been widely used to explore the partitioning and transport of photosynthesized C in plant shoots, roots, soil and soil microorganisms (Oikawa et al., 2017; Carmona et al., 2020). Photosynthesized C sequestration in farmland soils is strongly dependent on crop species and growth period. By integrating and analysing the articles on rice ^13^CO_2_ pulse labelling, Liu et al. (2019b) found that the distribution of photosynthesized C in rice shoots, roots and soil was 79%, 13% and 5.5%, respectively. Meng et al. (2013) conducted pulse labeling at the seedling, elongation, tassel and filling stages of maize and showed that the highest percentage of ^13^C was 27% and 3.8% in the roots and soil, respectively, during the elongation stage, while the lowest percentage was 3% and 2%, respectively, during the filling stage. By labelling soybean plants with ^13^CO_2_ at different growth stages, Jin et al. (2011) observed that 7.5% and 71.1% of photosynthesized C was fixed in the aboveground portion of soybean at the V4 and R6 stages, respectively, in comparison to in the belowground portion, where the proportion of photosynthesized C in the soil decreased significantly with fertility. Previous studies on the crop C cycle have mostly focused on major grains, but little research has been conducted on the transport and allocation of photosynthesized C in the economic crop sugarcane. Sugarcane is the most important sugar crop in China and is widely planted in South China on approximately 170 hectares, and its production is related to the country’s livelihood (Ou et al., 2013).

Sugarcane is a high N-demanding crop, with sufficient N being the basis for high yield, while N fertilization also affects the fate of photosynthesized C in the plant-soil system (Otto et al., 2021; Zhang et al., 2022). Increasing N fertilization not only promotes plant growth but also increases the chlorophyll content in the leaves and thus the photosynthesized rate of the plant, which further affects the input of photosynthesized C into the plant and its transport to roots and soil (Padilla et al., 2018; Wang et al., 2019; Zhao et al., 2019). The effectiveness of N in soils can also affect the mineralization of photosynthesized C in rhizosphere sediments by influencing microbial community diversity and abundance, which further affects C sequestration in soils (Ge et al., 2017; Pausch and Kuzyakov, 2018).

A large amount of N fertilizer invested to maintain sugarcane production often negatively affects the ecological environment (Alavi et al., 2017; Thorburn et al., 2017). For example, excessive use of N fertilizer leads to the leaching of N into groundwater which could promote the volatilization of NO, N_2_O, and NH_3_. The production of these gases pollutes local air and, at the same time, creates strong positive feedback on regional and global warming (Robertson et al., 2013; Qiao et al., 2015). How inhibiting nitrification affects nitrogen cycle and reduces environmental impacts of anthropogenic nitrogen input. Global Change Biology. 21, 1249-1257.To reduce the application of chemical N fertilizer to balance sugarcane production and environmental protection, the mixed planting of N-fixing crops and sugarcane has become a feasible planting pattern (Hoogmoed et al., 2014). Soybean, a high-quality N-fixing crop, is widely used in intercropping with sugarcane to reduce the use of chemical N fertilizer (Luo et al., 2016; Liu et al., 2021). Research shows that in native ecosystems, to compete with their neighbors, plants usually adopt a strategy of putting more C into functional organs (such as roots and shoots) to obtain limited resources, and plant diversity affects the belowground allocation of photosynthesized C (Fan et al., 2008; Trugman et al., 2019). Interspecific complementarity and competition also exist among different crops in intercropping systems. Aboveground complementarity promotes light interception and utilization efficiency through differences in crop height and light requirements (Zhang et al., 2008; Yu et al., 2015). Belowground complementarity increases water and nutrient access through ecological niche differentiation and resource allocation. In addition to the complementarity between different crops, competition for light, water and nutrients is inevitable because of limited resources (Duchene et al., 2017; Hu et al., 2021). Both the complementary and the competitive resources between sugarcane and soybean induce changes in photosynthesized C allocation aboveground and input belowground. In addition, Lian et al. (2019) showed that sugarcane/soybean intercropping changed soil microbial functions and promoted the C sequestration function of soil prokaryotes. Our previous study also indicated that long-term sugarcane/soybean intercropping with reduced N fertilization not only increased crop productivity but also reduced C footprint (Wang et al., 2020).

We already know that both intercropping patterns and N fertilizer additions affect crop growth and photosynthesized C sequestration in the soil. However, the partitioning and translocation of photosynthesized C in the plant-soil system in the sugarcane/soybean intercropping pattern and its potential mechanisms are not clear. Therefore, in this study, we aimed to 1) quantify the allocation of photosynthesized C in shoots, roots, and soil in sugarcane/soybean intercropping pattern; 2) track distribution of recently fixed C in the soil microbial C pools; and 3) explore the mechanism underlying cropping pattern and N fertilization addition driven photosynthesized C sequestration in the soil.

## 2 Materials and methods

### 2.1 Soil preparation

Soil samples were collected from the tillage layer (0-30 cm) of a long-term sugarcane experimental field (2009-2022) location at the experimental station of South China Agricultural University, Guangdong Province, China (23°8′N, 113°15′E) (Tang et al., 2017; Yu et al., 2019). The basic characteristics of the soil are as follows: pH 6.3, organic matter 12.57 g/kg, total N 0.66 g/kg, total P 0.69 g/kg, total K 18.61 g/kg, available N 58.25 mg/kg, available phosphorus 81.10 mg/kg, and available potassium 27.98 mg/kg. The δ^13^C value of the soil organic C (SOC) is -16.956 ± 0.2‰. Moist soil was homogenized and sieved (< 4 mm), and fine roots and other plant residues were manually removed.

### 2.2 Experimental design

A pot experiment was conducted in July 2022 in a randomized complete block design with two factors (i.e., cropping pattern and N addition level). For the cropping patterns, two sugarcane and soybean cropping system patterns were implemented: (1) sugarcane monoculture (MS); (2) sugarcane/soybean intercropping (SB). Similarly, N addition levels were calculated based on N application strategies from long-term field trials with tillage layer thickness and soil capacitance (Yang et al., 2013). They were reduced N (N1: 0.2 g·kg^-1^) and conventional N (N2: 0.4 g·kg^-1^). The experiment involved 4 treatments with 16 replicates for each treatment. The size of the pots used in the experiment was 30 cm long × 20 cm width× 15 cm height, and each pot was filled with 10 kg of sieved soil and set aside in a shaded area. Then sugarcane tubers and soybean seeds were placed in seedling trays to start seedlings on July 5 and 28, 2022, respectively, and then sugarcane and soybean seedlings were transplanted into pots on August 5. Specifically, two sugarcane seedlings were planted in each pot in the sugarcane monoculture pattern, and one sugarcane seedling and one soybean seedling were planted in sugarcane/soybean intercropping pattern. Sterile water was poured every day, soil moisture was kept between 15% and 25%, and the position of the plastic basin was changed every 7 days to reduce the error caused by uneven light and heat.

### 2.3 ^13^C pulse labelling

**Figure 1:**
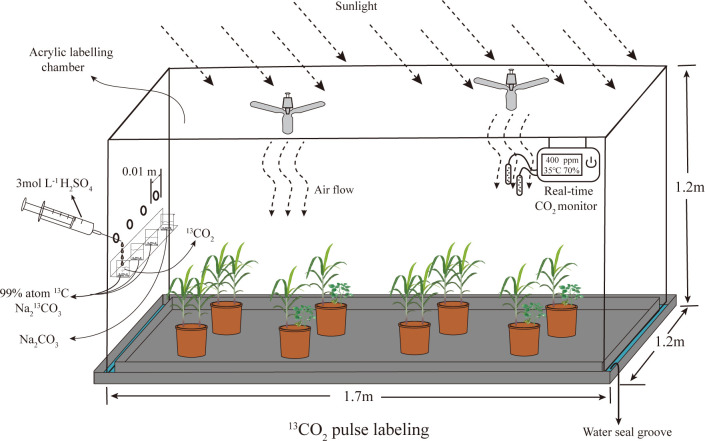
The structure of ^13^C labeling chamber.

The ^13^C pulse labelling experiment was carried out at the seedling stage of sugarcane. In each treatment, 8 pot plants were randomly selected for ^13^C labelling, and an additional 8 pot plants were selected for natural abundance δ^13^C. A chamber (1.7 m long×1.2 m width ×1.2 m heigh) constructed of acrylic (4 mm thickness) adapted from (Ge et al., 2015) was used for ^13^CO_2_ labelling (**Figure 1**). ^13^CO_2_ was generated through a reaction between 4.68 g Na_2_>^13^CO_3_ (99 atom% ^13^C, Sigma-Aldrich) and 50 ml of 3 M H_2_SO_4_ to obtain a ^13^CO_2_ concentration of approximately 400 ppm. Two fans were installed diagonally at the top of the marking chamber to ensure an even distribution of ^13^CO_2_. The labelling process started at 8:00 am on a sunny day and lasted for 6 h. The surface of the potting soil was covered with plastic wrap and sealed with silicone, including around the plant stems, before labelling to prevent ^13^CO_2_ from entering the soil directly (Kuzyakov et al., 2002). A real-time CO_2_ detector (Beijing Analytical Equipment Co.) was connected to the chamber to monitor the total CO_2_ concentration in the chamber (Wu et al., 2009). Pure air (without CO_2_) was introduced into the chamber to rapidly reduce the CO_2_ concentration. After the CO_2_ concentration fell below 50 ppm, H_2_SO_4_ (50 ml, 3 M) was added to the first beaker containing labelled Na_2_^13^CO_3_. When the CO_2_ concentration in the chamber fell below 50 ppm again, H_2_SO_4_ (50 ml, 3 M) was added to the 2nd beaker containing labelled Na_2_^13^CO_3_. This process was repeated three times. Finally, the same amount of H_2_SO_4_ was added to the fourth beaker containing unlabelled Na_2_^12^CO_3_ to enhance the ^13^C assimilation efficiency (An et al., 2015). The plants were removed from the chamber after the CO_2_ concentration dropped below 50 ppm.

### 2.4 Plant and soil sampling

Plants and soil were destructively sampled 1 day and 27 days after ^13^CO_2_ labelling. At each sampling, shoots were cut along the soil surface, and then, the roots and soil were separated by vibration (Zang et al., 2019). The soil samples were divided into two parts, one part was stored in a refrigerator at 4°C for the determination of microbial biomass C (MBC) and dissolved C (DOC) and the other naturally air-dried at room temperature and passed through a 2 mm sieve for the determination of soil total organic C (SOC), ^13^C abundance and soil physical and chemical properties (Zhu et al., 2017b). All shoot and root samples were dried in an oven at 70°C for 48 hours, weighed, ground with a ball mill and passed through a 0.15 mm sieve to determine plant total C and ^13^C abundance.

### 2.5 Measurement of soil MBC and DOC

Soil MBC was determined from fresh soil by the chloroform-fumigation extraction method (Brookes et al., 1985). The equivalent of 10 g of oven-dried fresh soil was fumigated for 24 h, followed by the addition of 40 ml of 0.5 M K_2_SO_4_ and shaking at 250 rpm for 1 h, and the final solution was then filtered using a 0.45 μm filter membrane to obtain the test solution. Similarly, the same amount of soil was extracted without fumigation. The nonfumigated extract was used to determine DOC in the soil. The soil extracts were measured to determine the dissolved organic C content using a total organic C analyser (Element high TOC II, Germany). The MBC was calculated as the difference in the total organic C content between fumigated and nonfumigated soil extracts and corrected using a conversion coefficient (k_EC_) value of 0.45 (Wu et al., 1990). The K_2_SO_4_ extracts were freeze-dried to analyze ^13^C abundance. The soil, plant, and solution materials were analyzed for TOC content and ^13^C abundance using a total organic C analyser (Element high TOC II, Germany) and an isotope ratio mass spectrometer (IsoPrime 100 Isotope Ratio Mass Spectrometer, Germany), respectively.

### 2.6 Isotopic C analysis and calculations

The amount of ^13^C incorporated into each part (shoot, root and soil) in the plant-soil system was calculated from the following equation (Leake et al., 2006):


(1) $${\;}^{13}\text{C conten}{\text{t}}_{\left(g/kg\right)}=\text{C conten}{\text{t}}_{\left(g/kg\right)}\times {(}^{13}{\text{C}}_{atom\%\;labelled}{-}^{13}{\text{C}}_{atom\%\;unlabelled})$$

where C content refers to the C content in the samples; ^13^C*_atom%_*_labelled_ and ^13^C*_atom% unlabelled_* refer to the ^13^C*_atom%_* of the labelled sample and unlabelled sample, respectively. The ^13^C*_atom%_* was calculated as (Lu et al., 2002):


(2) $${\;}^{13}{\text{C}}_{atom\%}=[(\delta^{13}\text{C}+1000)\times {\text{R}}_{PDB}]\times 100/[(\delta^{13}\text{C}+1000)\times {\text{R}}_{PDB}+1]$$

where R*_PDB_*_is_ equal to 0.011802 (Werner and Brand, 2001).

The proportion of ^13^C incorporated into each compartment of the plant-soil system was expressed as follows:


(3) $${\;}^{13}{\text{C}}_{proportion}(\%){=}^{13}{\text{C}}_{fixed}{/}^{13}{\text{C}}_{total\;fixed}$$

where ^13^C*_fixed_* refers to the fixed amount of ^13^C in each compartment, and ^13^C*_total fixed_* refers to the sum amount of ^13^C in each compartment.

The ^13^C content in the MBC (^13^C-MBC) was calculated with the following equation (Lu et al., 2002):


(4) $$\begin{array}{c} {\;}^{13}{\text{C}}_{content}\;MBC=[{(}^{13}{\text{C}}_{atom\%\;fum\;labelled}{-}^{13}{\text{C}}_{atom\%\;fum\;unlabelled})\\ \times {\text{C}}_{fum}-{(}^{13}{\text{C}}_{atom\%\;unfum\;labelled}\\ {-}^{13}{\text{C}}_{atom\%\;unfum\text{nbsp;}unlabelled})\\ \times {\text{C}}_{unfum}]/100/0.45 \end{array}$$

where “fum” and “unfum” refer to the “fumigated” and the “unfumigated” K_2_SO_4_ extracts, respectively, and C*_fum_* and C*_unfum_* represent the amounts (mg·kg^−1^ soil) of fumigated and unfumigated K_2_SO_4_ extracts, respectively.

### 2.7 Statistical analysis

Before data analysis, normality and homogeneity of variance were evaluated for all the data through Shapiro-Wilk tests and Levene’s tests using SPSS 20.0 (SPSS Inc., Chicago, IL, USA), respectively. One-way analysis of variance (ANOVA) combined with Fisher’s least significant difference (LSD) method was used to test for significant differences between treatments, with *p<* 0.05 considered a statistically significant difference. To determine the key drivers of the photosynthesized C sequestration in soils, we first conducted a Pearson correlation analysis using the vegan R package to determine the relationship between soil physicochemical properties, soil labile organic C and photosynthesized C sequestration. Based on the key drivers, the partial least squares path model (PLS-PM) was used to identify the association with photosynthesized C sequestration (Ren et al., 2022). Pearson correlation analysis and PLS-PM were conducted using R statistical software v.4.0.2. All charts were prepared using OriginPro 2020 (Origin Lab Corporation).

## 3 Results

### 3.1 Shoot, root biomass, and root/shoot ratio

**Figure 2:**
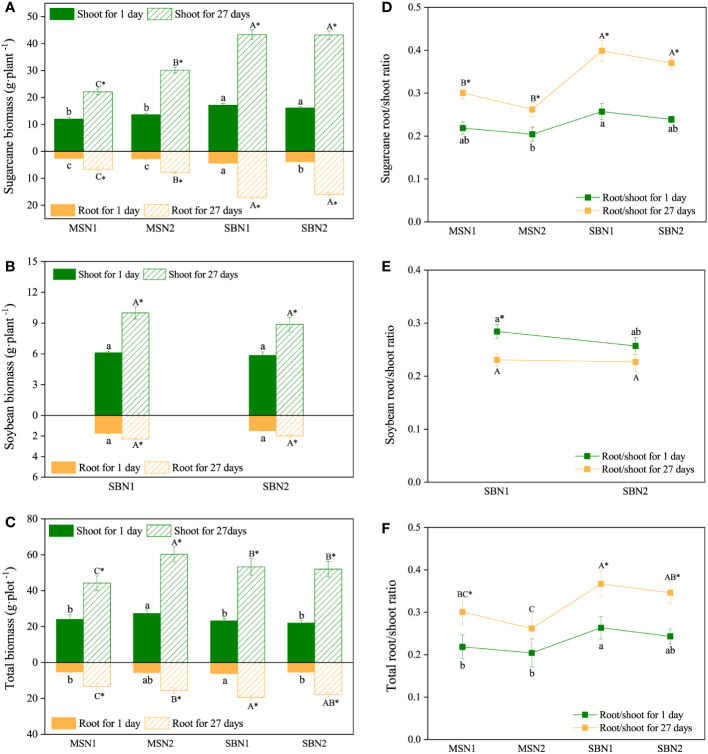
Effects of cropping pattern and N addition levels on the biomass and root/shoot ratio of sugarcane **(A, D)**, soybean **(B, E)** and the total ratio **(C, F)** on days 1 and 27 after labeling. Different letters represent significantly different means (p< 0.05) between treatments (lowercase for 1 day; uppercase for 27 days). An asterisk (*) denotes significantly different means (*p*< 0.05) between days.

The shoot and root biomass of the sugarcane/soybean intercropping system increased compared to that of the sugarcane monoculture on days 1 and 27 after labeling (*p<* 0.05). N addition only increased monocropping-sugarcane (sugarcane per plant in sugarcane monoculture systems) biomass, but had no significant effect on intercropping-sugarcane (sugarcane per plant in sugarcane/soybean intercropping system) biomass (**Figure 2A**). In contrast to sugarcane, soybean (soybean per plant in sugarcane/soybean intercropping systems) biomass was not affected by the N addition level (**Figure 2B**). For total biomass, the maximum shoot biomass in the MSN2 treatment was 27.30 and 60.29 g·plot^-1^ on days 1 and 27 after labeling, respectively. Interestingly, the total root biomass of the sugarcane/soybean intercropping pattern was significantly higher than that of the sugarcane monoculture system under both low and high N conditions on day 27 after labeling (**Figure 2C**).

Compared with day 1 after labeling, both root/shoot and total ratios increased in sugarcane and decreased significantly in soybean at day 27 after labeling (**Figures 2C, D, F**). Specifically, the root/shoot ratio of intercropping-sugarcane was significantly higher than that of monocropping-sugarcane (**Figure 2D**). The total root/shoot ratio in the sugarcane/soybean intercropping system was higher than that in the sugarcane monoculture patterns on days 1 and 27 after labelling (**Figure 2F**). We also observed that the N addition amount had no significant influence on the root/shoot ratio in either the monoculture or intercropping patterns (**Figures 2D–F**).

### 3.2 ^13^C allocation amount and proportion in the plant-soil system

**Figure 3:**
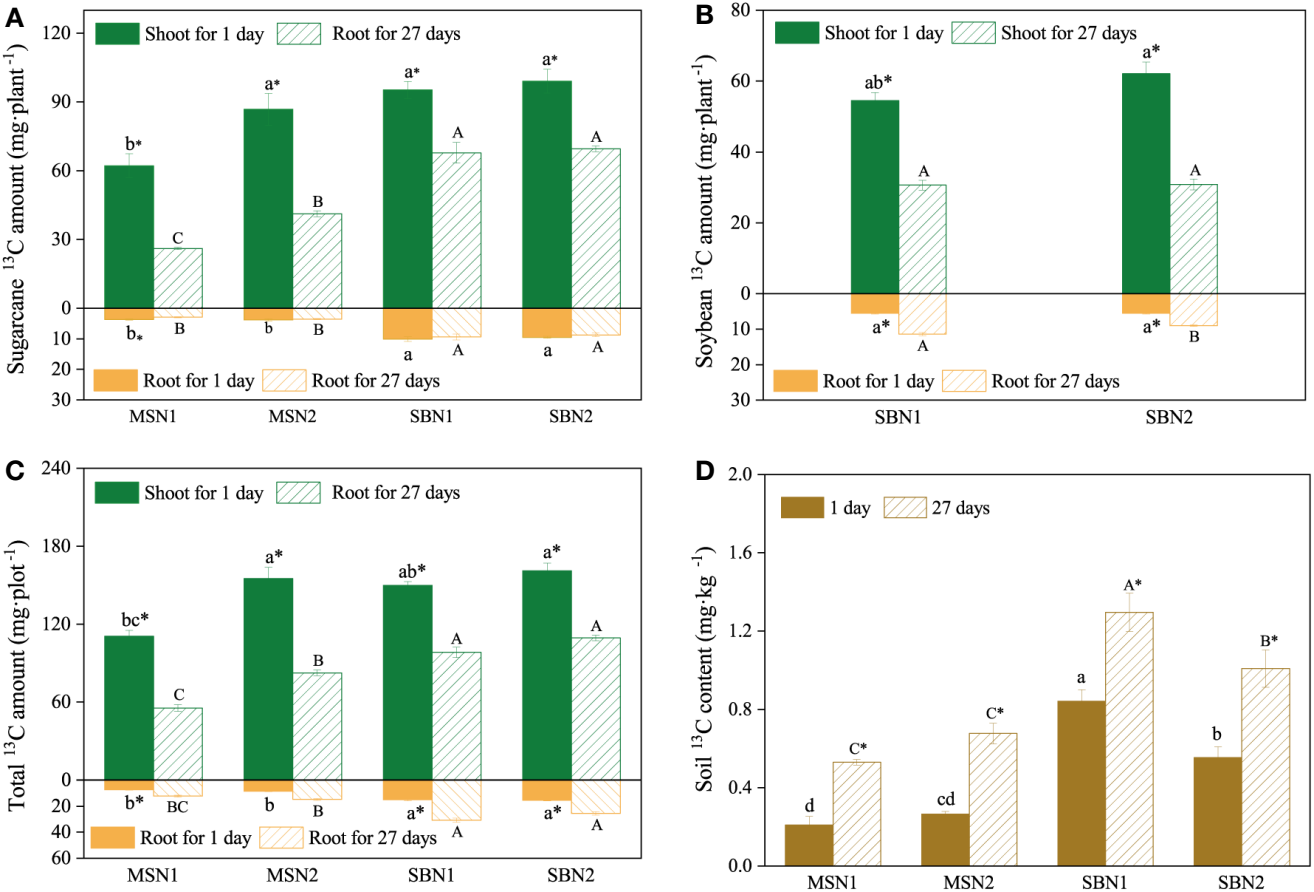
Effects of cropping pattern and N addition levels on the ^13^C amount of sugarcane **(A)**, soybean **(B)**, total **(C)** and the soil **(D)** ^13^C content on days 1 and 27 after labeling. Different letters represent significantly different means (p< 0.05) between treatments (lowercase for 1 day; uppercase for 27 days). An asterisk (*) denotes significantly different means (*p*< 0.05) between days.

**Figure 4:**
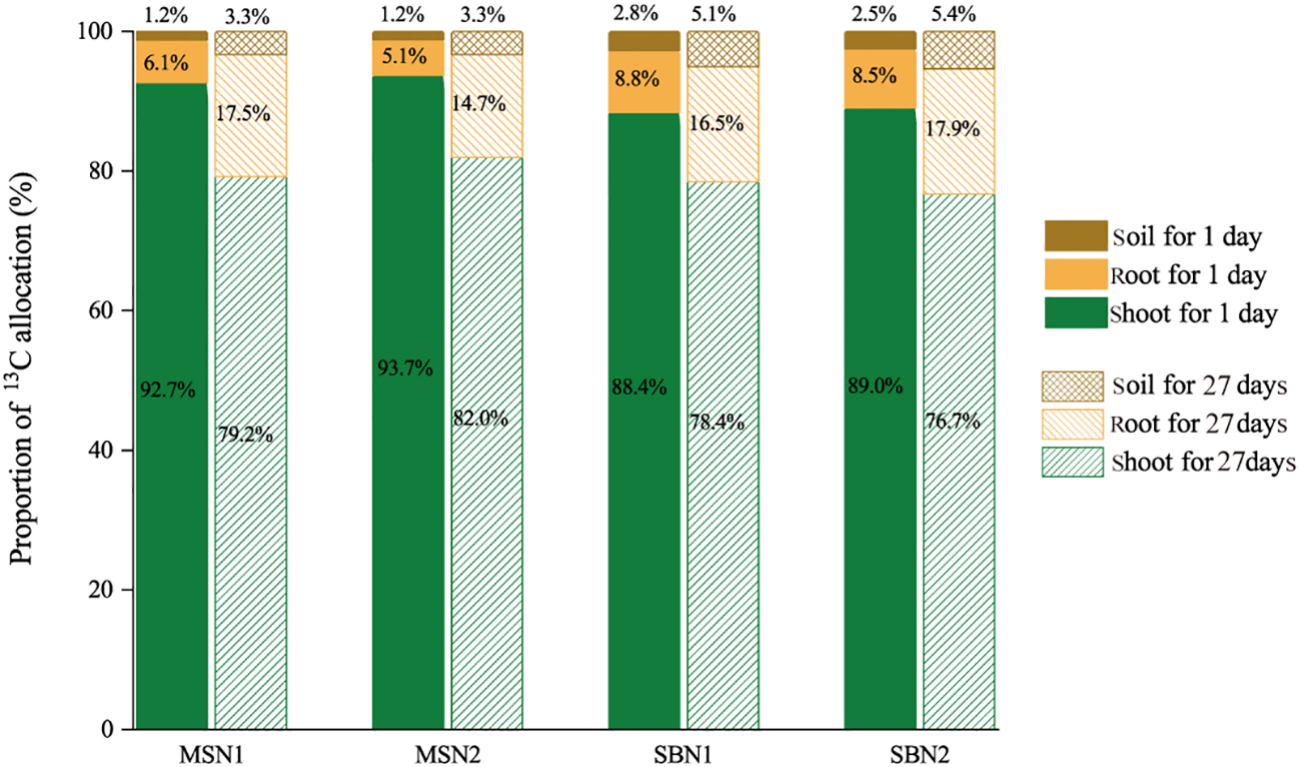
Effects of cropping pattern and N addition levels on the shoot, root and soil ^13^C allocation proportion (%) on days 1 and 27 after labeling.

As shown in **Figures 3** and **4**, compared with day 1 after labelling, the ^13^C amount and proportion in the shoots decreased significantly, and the ^13^C amount and proportion in the soil increased significantly on day 27 after labeling, which indicates that ^13^C was transferred from the shoots to the roots and soil. More specifically, after days 1 and 27 after labelling, compared to monocropping-sugarcane, except for the MSN2 treatment after 1 day of labeling, intercropping-sugarcane significantly increased ^13^C amounts in the shoots and roots. The increase in the N addition amount only increased the shoot ^13^C amount of monocropping-sugarcane (**Figure 3A**). Although the total ^13^C amount in the shoots in the MSN2 treatment was significantly higher than that in all other treatments on day 1 after labeling, the total ^13^C amount in the roots was significantly lower than that in the roots in the sugarcane/soybean intercropping pattern. On day 27 after labeling, compared with sugarcane monoculture pattern, the total amount shoot ^13^C and root ^13^C in the sugarcane/soybean intercropping pattern increased by 47.74% and 120.85%, but the shoot ^13^C proportion decreased by 3.74% (**Figures 3C**, **4**). Similarly, we observed that the sugarcane/soybean intercropping pattern promoted the fixation of ^13^C in the soil (**Figures 3D**, **4**). On days 1 and 27 after labeling, the soil ^13^C amount and proportion in the sugarcane/soybean intercropping pattern was significantly higher than that in the sugarcane monoculture pattern. In the sugarcane monocropping pattern, the increase in N application level did not result in a significant increase in both soil ^13^C content and proportion. However, in the sugarcane/soybean intercropping pattern, soil ^13^C content and proportion increased by 28.44% and 56.12%, respectively, at the N1 level compared to the N2 level at 27 days after ^13^CO_2_ labelling.

### 3.3 Content of ^13^C-MBC, ^13^C-DOC and the ratio of ^13^C-MBC/^13^C-SOC, ^13^C-DOC/^13^C-SOC

**Figure 5:**
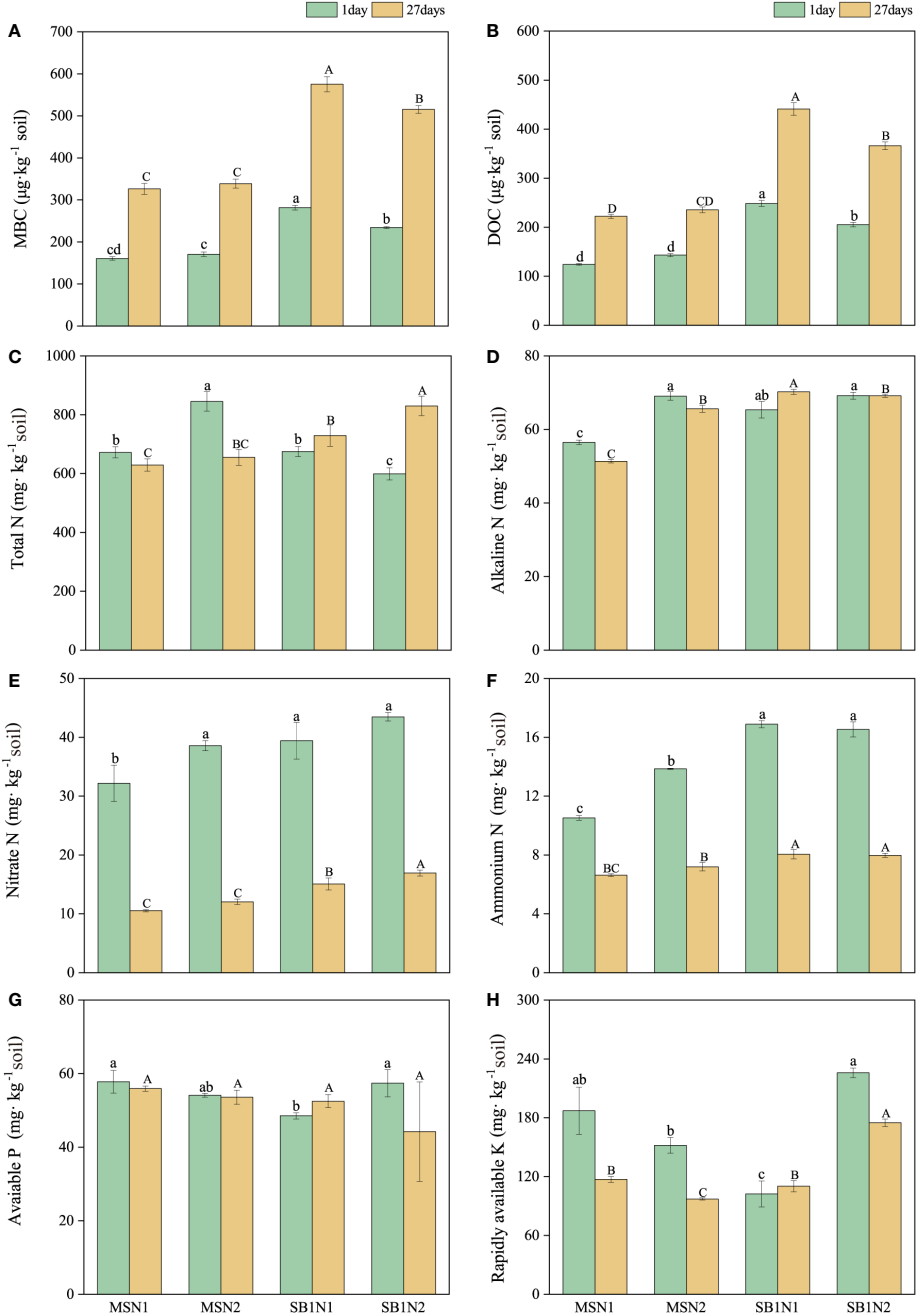
Effects of cropping pattern and N addition levels on the soil physicochemical properties **(A–H)** on days 1 and 27 after labeling. Different letters represent significantly different means (*p*< 0.05) between treatments (lowercase for 1 day; uppercase for 27 days).

The content of soil ^13^C-MBC and ^13^C-DOC in the soil showed a decreasing trend from day 1 to day 27 after labeling (*p<* 0.05). On days 1 and 27 after labeling, the content of soil ^13^C-MBC and ^13^C-DOC in the sugarcane/soybean intercropping pattern was significantly higher than that in the sugarcane monoculture pattern. In contrast, in the intercropping pattern, the contents of ^13^C-MBC and ^13^C-DOC increased by 13.55% and 12.92% at day 1 after labelling and by 14.56% and 10.12% on day 27 after labeling at N1 level compare to those at N2 level. (**Figures 5A, B**). Similar to the temporal trend of the MBC and DOC content, the ratio of ^13^C-MBC/^13^C-SOC and ^13^C-DOC/^13^C-SOC also showed a decreasing trend from day 1 to day 27 (**Figures 5C, D**). The difference was that the cropping pattern and N addition level did not significantly affect the ratios of ^13^C-MBC/^13^C-SOC and ^13^C-DOC/^13^C-SOC.

### 3.4 Soil physicochemical properties

The content of MBC and DOC in the soil showed a significantly increasing trend from day 1 to 27 after labelling. On days 1 and 27 after labeling, the content of soil MBC and DOC in the sugarcane/soybean intercropping pattern was significantly higher than that in the sugarcane monoculture pattern. In contrast, the increase in the N addition level in the sugarcane/soybean intercropping treatment significantly decreased the content of soil MBC and DOC (**Figures 5A, B**). The soil total N content of the MSN2 treatment was the highest at 1 day after labelling, but at 27 days after labelling, compared to MSN2 treatment, SBN1 and SBN2 treatments increased by 11.3% and 26.72%, respectively (**Figure 5C**). Nitrate and ammonium N contents were significantly higher in the sugarcane/soybean intercropping pattern than in the sugarcane monocropping pattern at both day 1 and day 27 after labeling (**Figures 5E, F**). The soil alkaline N content of the MSN1 treatment was significantly lower than that of the other treatments (**Figure 5D**). In terms of soil available phosphorus (P) and available potassium (K), the available K content in the SBN1 treatment was higher than that in the other treatments, while the available P content in each treatment was not substantially different (**Figures 5G, H**).

### 3.5 Linkage of soil physicochemical properties with ^13^C sequestration in soil

**Figure 6:**
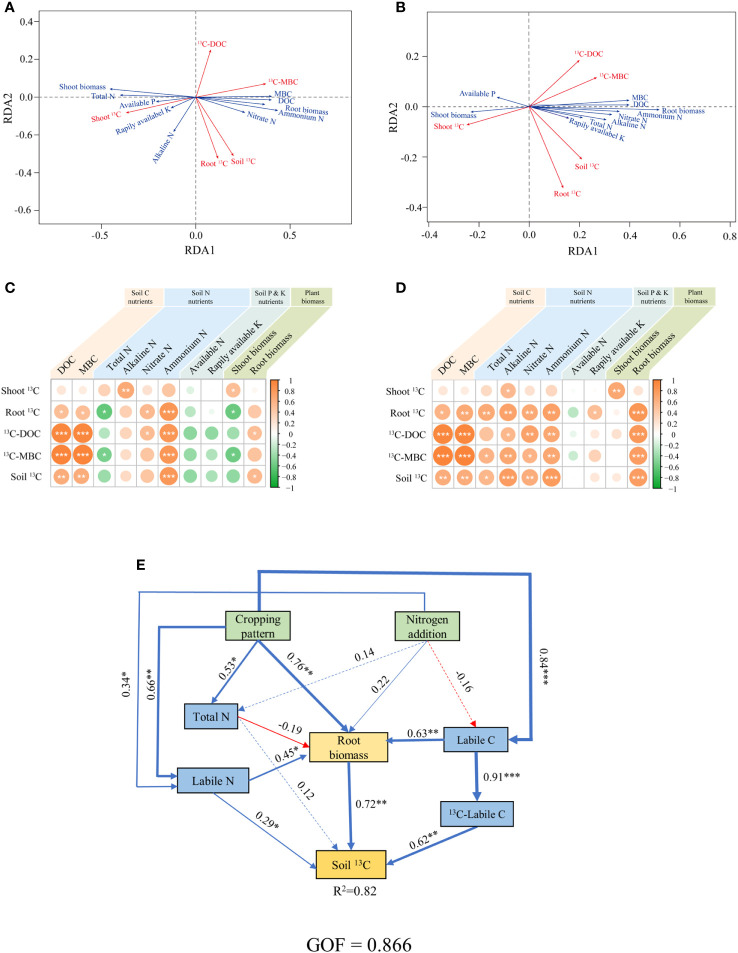
**(A–D)** The relationships among ^13^C contents in each compartment of the plant-soil system, and soil physicochemical and plant biomass on days 1 **(A, C)** and 27 **(B, D)**. **(E)** Structural equation model showing the factors regulating the ^13^C transfer to soil in the plant-soil system, the data collected on day 27 after labeling. The red and blue arrows accordingly denote the negative and positive pathways. LOC: dissolved organic C and microbial biomass C; TN: total N; LN: Nitrate N, ammonium N and alkaline N. *p< 0.05, **p< 0.01, ***p< 0.001.

According to Pearson correlation and RDA analysis, at day 1 after labelling, the content of DOC, MBC and ammonium N in the soil were positively correlated with the content of photosynthesized C in soil labile C (^13^C-Labile C) and in soil total organic C (Soil ^13^C) (*p*< 0.05) (**Figures 6A, C**). And on day 27 after labeling, the content of alkaline N, nitrate N and root biomass were also positively correlated with Soil ^13^C content (*p*< 0.05) (**Figures 6B, D**). We used structural equation modelling to further explore how cropping pattern and N addition driven soil physicochemical properties to affected photosynthesized C sequestration in soil (**Figure 6E**). The results show that cropping pattern positively affected soil labile N, soil labile C and root biomass, where both soil labile C and N positively influenced root biomass, and soil labile C also affected ^13^C-labile C. Finally, soil labile N, ^13^C-labile C and root biomass jointly positively affected soil ^13^C content, and explained a high percentage (82%) of the variance in soil ^13^C content (*p<* 0.05; **Figure 6E**).

## 4 Discussion

### 4.1 Allocation of photosynthesized C in the plant-soil system

**Figure 7:**
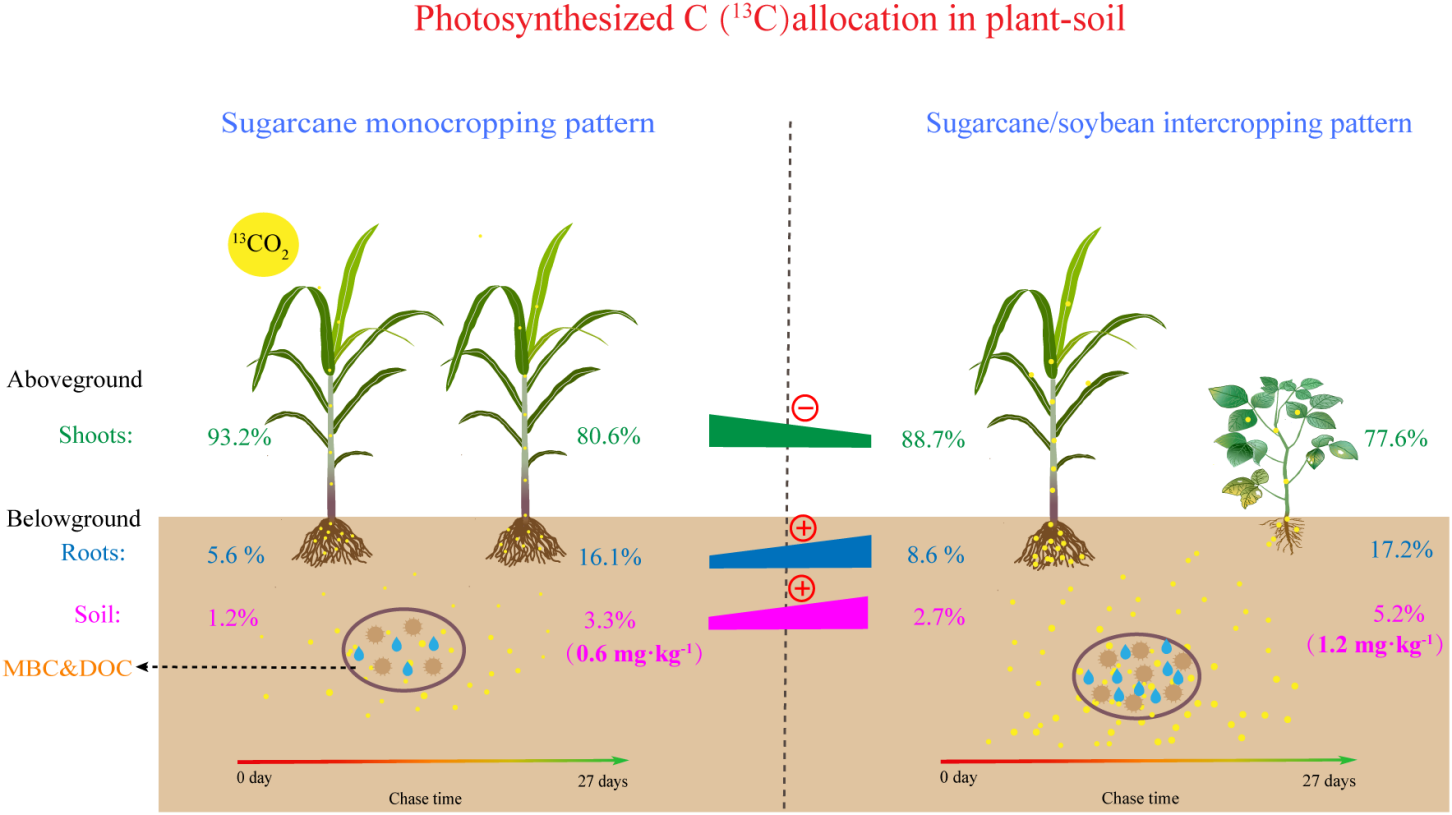
Schematic diagram depicting the impacts of intercropping on photosynthesized carbon allocation in plant-soil system.

**Figure 8:**
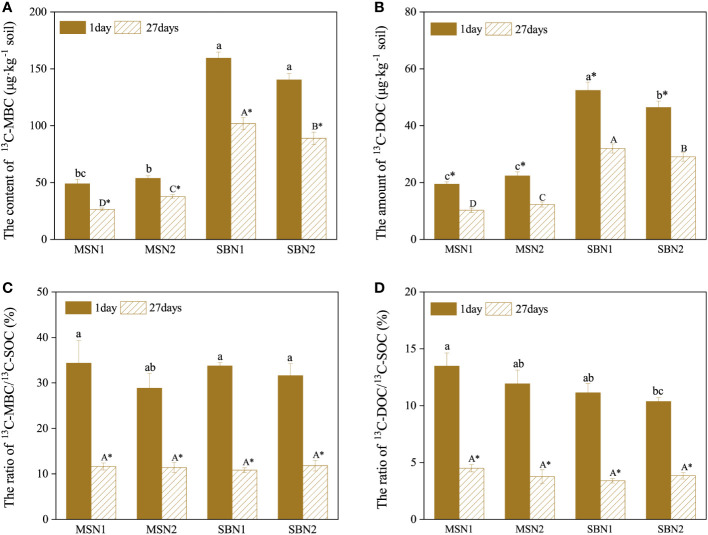
Effects of cropping pattern and N addition levels on the content of soil ^13^C-MBC, ^13^C-DOC **(A, B)** and the ratio of ^13^C-MBC/^13^C-SOC, ^13^C-DOC/^13^C-SOC **(C, D)** on days 1 and 27 after labeling. Different letters represent significantly different means (*p*< 0.05) between treatments (lowercase for 1 day; uppercase for 27 days). An asterisk (*) denotes significantly different means (*p*< 0.05) between days.

In the last decade, considerable attention has been given to the process of photosynthesized C partitioning and translocation in plant-soil in agroecosystems (Mo et al., 2019; Zhao et al., 2021). N addition, as an important agronomic practice in agricultural production, strongly influences crop growth and development, and crop biomass is directly related to the fixed of photosynthesized C in plants and soil (Liu et al., 2014; Zhu et al., 2017a). We found that both sugarcane plants and soils in MSN2 treatment fixed more photosynthesized C than MSN1 treatments (**Figure 3**). The increase in photosynthesized C fixation in both the crop and soil was mainly due to the increase in sugarcane biomass promoted by the increased N addition (**Figure 1A**). This result is similar to the findings of Ge et al. (2017), who found that the amount of photosynthesized C in both DOC and SOC increased with increased N addition by studying photosynthesized C sequestration in the paddy soil at four N fertilizer levels. However, we noticed that photosynthesized C sequestration in sugarcane and soybean plants were not significantly different N addition levels (**Figures 3A, B**). This result probably occurred because in the sugarcane/soybean intercropping pattern, sugarcane can utilize the N fixed from the atmosphere by soybean, reducing the dependence of sugarcane on chemical N. Therefore, the reduced N addition did not inhibit sugarcane and soybean growth and photosynthesized C fixation (Tian et al., 2020). In addition, photosynthesized C fixation in the soil was significantly higher in the N1 treatment than in the N2 treatment (**Figure 3D**). This may be attributed to the fact that in the sugarcane/soybean intercropping pattern, the microorganisms in the soil were more numerous and more active at low N levels, thus allowing more photosynthesized C to be sequestered using more rhizodeposits (Chen et al., 2019). Moreover, photosynthesized C sequestration in both plant and soil was significantly higher in the sugarcane/soybean intercropping pattern than in the sugarcane monoculture pattern (**Figures 3**, **7**). Sugarcane, the dominant species in the sugarcane/soybean intercropping pattern, competes for more nutrients and water from the soil by increasing root biomass and area of distribution, and also absorbs N fixed from the air by neighboring soybeans through the roots and mycelium network. The increase in shoot biomass of sugarcane was supported by the adequate resources obtained by the roots, and the good development of the shoot naturally leads to more sunlight and higher photosynthetic capacity of the leaves, which in turn promotes the fixation of more photosynthesized C in the aboveground of the plant (Wang et al., 2020). In addition, the more abundant roots and mycelium network in the sugarcane/soybean intercropping pattern can also transfer more photosynthesized C to the soil. A large portion of the photosynthesized C entering the soil is mineralized as CO_2_ and released to the atmosphere, while only a portion of the photosynthesized C is finally sequestered in the soil (Liu et al., 2023). The efficiency of photosynthesized C sequestration in soil is driven by a combination of abiotic and biotic factors, the most critical of which are soil microorganisms. Microbial necromass has been reported to contribute 15-80% to soil organic C (Angst et al., 2021). They are responsive to exogenous organic matter inputs as key players in soil organic C transformation processes and can utilize photosynthesized C in rhizodeposits in the first instance (Niu et al., 2018). Microorganisms that absorb photosynthesized C then retain the residues in the soil after apoptosis to complete the sequestration of photosynthesized C. In general, the activity and abundance of microorganisms in the soil largely determine the efficiency of photosynthesized C sequestration in the soil. The results of this experiment support the idea that soil MBC content and photosynthesized C content were both highest under the SBN1 treatment (**Figures 3**, **8**).

### 4.2 Distribution of recently fixed C in the soil labile C pools

MBC is composed of microbial residues and is often used to indicate the abundance of microbial communities in soil (Xu et al., 2020). On day 1 after ^13^CO_2_ labelling, approximately 30% of photosynthesized C retained the soil was incorporated into MBC (**Figure 8C**). This result suggests that microbial metabolism preferentially utilized C in the root exudates, which consisted mainly of soluble compounds such as low molecular weight monosaccharides, organic acids, and amino acids (Kaiser et al., 2015; Starr et al., 2018). Photosynthesized C can be rapidly transferred from plant tissues to the soil, and some studies have shown that ^13^C can be detected in soil as early as 12 h after ^13^C labelling (Gavrichkova and Kuzyakov, 2017; Liu et al., 2019a). The rapid translocation of photosynthesized C resulted in lower ^13^C content in the rhizodeposits 27 days after labelling, and more ^13^C had been fixed in plant tissues in the form of lignin and cellulose, which are difficult to decompose. Therefore, ^13^C-MBC content was significantly lower at 27 days after labelling than at 1 day after labelling (**Figure 8C**) (Zhang et al., 2017). In addition, we found that the soil ^13^C-MBC content was significantly higher in the intercropping pattern than in sugarcane monoculture pattern, demonstrating that sugarcane/soybean intercropping increased the abundance and activity of microorganisms involved in photosynthesized C turnover, which could largely explain the efficiency of photosynthesized C sequestration in the soil (**Figure 8A**).

Both DOC and MBC are important indicators of labile C pool in soil and play an important role in the transformation of soil organic C (de Brito et al., 2019). However, the amount and proportion of ^13^C-DOC in the soil were much smaller than those of ^13^C-MBC (**Figures 8B, D**). DOC, as the sum of a range of dissolved C, can be preferentially used by microorganisms. A portion of the decomposed DOC is released into the air as CO_2_ or another portion is converted to more stable organic matter and stored in the soil, resulting in a lower ^13^C-DOC content in the soil. (Farrell et al., 2014). Our data showed that the ^13^C-DOC content was 101.85% higher in the sugarcane/soybean intercropping pattern than in the sugarcane monoculture pattern (**Figure 8B**). This outcome may have occurred based on the following factors: first, sugarcane/soybean intercropping pattern can increase sugarcane root biomass and soil microbial activity, thus accelerating the release and decomposition of rhizodeposits and promoting the accumulation of soil ^13^C-DOC (Tang et al., 2017). Second, the sugarcane/soybean intercropping pattern provided more DOC to soil microbes and reduced ^13^C-DOC depletion, and in fact, our data did show higher soil DOC content in the N2 treatment in the sugarcane monoculture pattern than in the other treatments (**Figure 5B**). Third, the mycelium network constructed between sugarcane and soybean roots in an intercropping pattern can promote the binding of ^13^C-DOC to soil particles to form larger soil aggregates, increasing the difficulty of exposing microbes to ^13^C-DOC (Sankaranarayanan and Hari, 2021).

### 4.3 Correlation of soil physicochemical properties with photosynthesized C sequestration in soil

Increasing soil C sequestration capacity is receiving improving attention as an effective way to improve soil fertility and mitigate global temperature rise (Ramesh et al., 2019). There are two main ways to boost soil organic C. The first pathway is to increase the input of aboveground organic C, in the present experiment, our results showed that that the input of photosynthesized C to the aboveground depends mainly on the root biomass (**Figures 6B, D**). The other way is to reduce soil organic C mineralization to increase the rate of photosynthesized C sequestration in the soil. Microorganisms, as decomposers in the soil, are key players in driving SOC transformation and are directly related to photosynthesized C sequestration in the soil (Chen et al., 2019). The present study also showed that photosynthesized C in microorganisms was highly significantly and positively correlated with photosynthesized C sequestered in the soil (**Figure 6E**). Both root biomass and soil microbial activity are influenced by soil physicochemical properties, with soil C and N nutrients having the strongest influence (**Figure 6**) (Li and Wu, 2018). Soil mineral N content, as one of the important components of soil physicochemical properties, provides the basic energy for microbial reproduction and root growth (Liu et al., 2019b). Moreover, microorganisms need to absorb mineral N after assimilating photosynthesized C to maintain a stoichiometric ratio of microbial biomass (Zhang et al., 2021b). The results of Li et al. (2022a) further indicated that there is a significant positive correlation between ammonium N, soil C: N and C-fixing bacterial community abundance. Similar results were found in the sugarcane/soybean intercropping pattern, where intercropping increased the labile N content of the soil, resulting in the overexpression of the prokaryotic C sequestration pathway and thus contributing to the increase in soil organic C (Lian et al., 2019).

Structural equation models show that cropping patterns can regulate photosynthesized C sequestration in the soil by affecting soil labile C and N content (**Figure 6E**). In addition to agroecosystems, the effect of cropping patterns on soil physicochemical properties has been found in other ecosystems. For instance, in an agroforestry ecosystem, Wu et al. (2017) investigated soil physicochemical properties under rubber tree/cocoa bean intercropping pattern found that intercropping promoted an increase in SOC and TN in the 0-10 cm soil. The effect of intercropping between cereal and legume crops on soil physicochemical properties is mainly attributed to the interaction of competition and promotion between crop roots (Yu et al., 2022). Competition is due to the limited resources in the soil and the overlap of crop niches. The dominant crops in an intercropping pattern will obtain more water and nutrients by increasing their root biomass and root distribution area, resulting in higher soil nutrients and water content around cereal crops than around legumes (Hong et al., 2022). In addition, the dominant crops can also affect the growth of adjacent crops through the allelopathy of rhizosphere exudates (Wang et al., 2021). The main chemosensory substances include phenolic compounds, terpenoids and nitrogen-containing chemosensory substances, which affect crop growth as well as soil physical and chemical properties (Macias et al., 2019). Facilitation primarily refers to the N supply of legume crops to cereal crops, that is, legume crops transfer N fixed from the atmosphere to cereal crops through roots, and N transfer leads to differences in mineral N concentrations in soil (Zhang et al., 2021a). For the rapid transfer of N, a mycelium network is formed between the roots of the cereal crop and the legume crop, and the distribution of mycelium network in the soil affects the formation of agglomerates and thus the physicochemical properties of the soil (Thilakarathna et al., 2016).

## 5 Conclusions

The sugarcane/soybean intercropping pattern increased total root biomass by promoting the growth of sugarcane shoot and increasing the root/shoot ratio. More roots facilitate the transport of photosynthesized C into the soil, and the photosynthesized C that flows into the soil is first used by microorganisms. On day 1 after labelling, approximately 30% of the retained ^13^C in the soil was incorporated into the MBC. On day 27 after labeling, photosynthetic C in MBC under SBN1 treatment was significantly higher than that in the other treatments, as well as in the soil. In addition, the sugarcane/soybean intercropping pattern improved soil physicochemical properties and increased soil labile C and N contents. Correlation analysis and structural equation modelling indicated that cropping pattern regulates photosynthesized C sequestration in soil by affecting labile C content, labile N content and root biomass. In summary, the SBN1 treatment both promoted photosynthesized C flow to the soil by increasing root biomass and increased microbial abundance and activity by improving soil physicochemical properties. The increase in photosynthesized C input and soil microbial activity together increased the photosynthesized C sequestration in the soil. However, this study did not identify the key microbial species involved in photosynthesized C transformation and fixation in soil. In order to gain a deeper understanding of the photosynthesized C conversion process in soil, we need to isolate the key microorganisms involved in photosynthesized C transformation in soil by DNA-SIP and high-throughput sequencing technologies in the future.

## Data availability statement

The raw data supporting the conclusions of this article will be made available by the authors, without undue reservation.

## Author contributions

TZ: Writing – review & editing, Conceptualization, Investigation, Writing – original draft. HT: Writing – review & editing, Data curation, Methodology. PP: Investigation, Writing – review & editing, Funding acquisition, Visualization. SG: Writing – review & editing. YL: Writing – review & editing, Investigation. YF: Writing – review & editing. JW: Writing – review & editing, Funding acquisition.
